# The Dashboard Vitals of Parkinson’s: Not to Be Missed Yet an Unmet Need

**DOI:** 10.3390/jpm12121994

**Published:** 2022-12-02

**Authors:** Kallol Ray Chaudhuri, Nataliya Titova, Mubasher A. Qamar, Iulia Murășan, Cristian Falup-Pecurariu

**Affiliations:** 1 Institute of Psychiatry, Psychology & Neuroscience, Department of Basic & Clinical Neuroscience, Division of Neuroscience, King’s College London, London SE5 9RT, UK; 2 Parkinson Foundation Centre of Excellence in Care and Research, King’s College Hospital NHS Foundation Trust, London SE5 9RS, UK; 3 Department of Neurology, Neurosurgery and Medical Genetics, Federal State Autonomous Educational Institution of Higher Education, N.I. Pirogov Russian National Research Medical University, The Ministry of Health of the Russian Federation, 117997 Moscow, Russia; 4 Department of Neurodegenerative Diseases, Federal State Budgetary Institution, Federal Center of Brain Research and Neurotechnologies, The Federal Medical Biological Agency, 117997 Moscow, Russia; 5 Faculty of Medicine, Transilvania University of Braşov, 500036 Brașov, Romania; 6 Department of Neurology, County Clinic Hospital, 500365 Brașov, Romania

## 1. Commentary

The vitals of Parkinson’s disease (PD) address the often-ignored symptoms, which are considered either peripheral to the central core of motor symptoms of PD or secondary symptoms, which, nevertheless, have a key role in the quality of life (QoL) and wellness of people with Parkinson’s (PwP) [1]. Unmet needs in PwP have recently been discussed, with many being related to motor symptoms and, specifically, non-motor symptoms (NMSs), which continue to pose a major challenge to PwP and their clinicians [2]. In addition, several other factors related to enablers of PD expression, progression, as well as co-morbidities and co-medication issues compound the wellness of PwP and we proposed all PwP to have a dashboard, whereby clinical assessment for these symptoms must be noted and managed as bespoke to the individual person, a key element in modern personalized medicine for PD [3,4].

**Figure 1:**
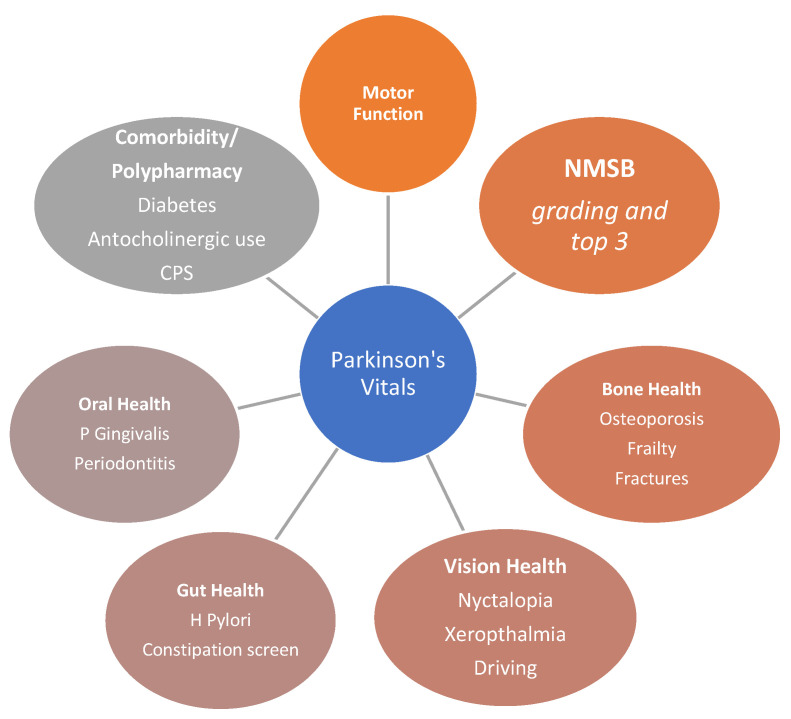
A diagram of the essential “vitals” to be considered in Parkinson’s disease which should form a dashboard of symptoms to be considered and managed in every person with Parkinson’s. NMSB non-motor symptom burden; H Pylori Helicobacter pylori; P Gingivalis porphyromonas gingivalis; CPS comorbidity polypharmacy score.

The key elements of the vitals to form a dashboard for PwP are shown in Figure 1. These include the essential motor assessment, which is completed in almost all clinics as the initial evaluation in consultations. Motor function can be graded by clinical examination and assigning the Hoehn and Yahr (H&Y) staging [5], which, despite its clinimetric drawbacks, continues to be the most widely used clinical assessment for tangible and real-life motor assessment of PD and has stood the test of time. If time permits and there is capacity, then detailed motor examinations are possible using the Scales for Outcomes in PD (SCOPA)-motor [6], Movement Disorder Society Unified PD Rating scale (MDS-UPDRS) [7], or even the older UPDRS parts 3 and 4 [8]. In the future, PD-validated wearable monitoring scores with sensors, such as Parkinson kinetograph (PKG), could be added [9,10].

Then, there is the burden of NMS assessments, which can be carried out and graded using either the validated NMS Questionnaire (NMS Quest) or, if time permits, utilizing the PD-NMS scale (NMSS) [11,12,13,14]. NMS burden (NMSB) should be performed for every patient and graded, alongside the patients and their caregivers, rating their top named bothersome NMS. NMSB is contributed to by a range of NMS, from cognitive issues, neuropsychiatric problems, such as depression, apathy, and anxiety, to sleep dysfunction, hyposmia, bladder, bowel, and upper gastrointestinal dysfunction, such as the dribbling of saliva. NMSB has a direct correlation with QoL and a guide to using the NMS Quest in the clinic has also been published. NMSB score should be integral to the dashboard and ideally measured on a yearly basis [15].

Vision is a critical aspect of living with PD and is rarely formally addressed in a PD clinic. A range of visual problems can occur in PD and these have been explored in several studies [16,17,18,19,20]. Vision assessment is important for PwP who continue to drive and, in this respect, night blindness (nyctalopia) and convergence insufficiency are important. Subsequently, a patient may have significant discomfort related to dry eyes (xeropthalmia), which is treatable with eye drops as well as glaucoma. The NMS Quest also allows for declaration of diplopia, which is common in PD and may be related to dyskinesias or convergence insufficiency. Nyctalopia may be related to vitamin A deficiency and may require night-time bedroom lighting to prevent falls at night-time should the patient need to get out of bed, for instance, to go to the toilet. Significant issues need a referral to an ophthalmologist [21].

Bone health is an integral aspect of Parkinson’s wellness and relates to a very high incidence of osteoporosis or osteopenia in PD and related risk of fractures with falls and frailty as well as subsequent risk of hospitalization. A global longitudinal study of osteoporosis in women, the GLOW study, reported PD to be the strongest and most robust contributor to risk of fractures compared with other studied factors [22]. Motor dysfunction, frailty, gait impairment and freezing, postural instability, diphasic or troublesome dyskinesias and falls, polypharmacy, and reduced bone density contribute towards the increased risk of fracture in PD [23,24,25,26]. Vitamin D deficiency along with disease duration and severity, age, and low body mass index (BMI) with secondary hyperparathyroidism may also contribute to low bone density and need to be evaluated in all PwP periodically and added to the dashboard [22].

When assessing PwP holistically, the issue of weight is often ignored in clinical consultations, although blood pressure, height, and weight are often routinely collected in the clinic. Low body weight poses a specific challenge in PD and a low body weight phenotype in PD, the Park-weight phenotype, has been proposed to have a high risk of dyskinesias, as well as possible links with cognitive dysfunction and hyposmia [27,28,29]. Weight and BMI, therefore, need to be noted at baseline in all PD cases and routinely charted for monitoring. Unexplained weight loss is a question asked in the NMS Quest and, in addition, may be a problem with some medications, such as intrajejunal levodopa infusion, as well as those with severe dyskinesias. Unexplained weight loss coupled with rising frailty has also been linked to future cognitive dysfunction and, therefore, also may have prognostic consequences [30,31].

Gut and oral health is another important enabler of wellness and health in PD and constitutes the important “vital” aspect for the dashboard. Gut dysfunction in PD is well documented and ranges from upper gastrointestinal dysfunction, such as dysphagia and delayed gastric emptying, to constipation [32].

While many of these symptoms are flagged up in the NMS Quest and constitute part of the NMSB, some need key and focused attention as they are often ignored in clinics. These include:

1. Specific attention and query about oral health, gum, and gingivitis and an examination by a dentist in all cases. Infection with porphyromonas gingivalis, a Gram-negative anaerobic bacterium, can cause chronic periodontitis and possibly systemic inflammation, together with gingipains, and may have an overall effect on worsening of the Parkinsonian state and even pathogenesis [33]. A recent study suggested that high serum C-reactive protein (CRP) level may be a good indicator of periodontitis and should trigger a referral to a dentist and needs to feature in the dashboard [34].
2. Delayed oral drug absorption as well as clinical phenomena of “delayed on” or “no on” or even dyskinesias-related erratic absorption may relate to delayed gastric emptying and “gastric blocks”. Helicobacter pylori (H Pylori) infection, a Gram-negative bacteria, in the stomach is common in PD and several case-control studies report that prevalence of H Pylori infection is five-times higher in older PD patients, specifically those over 80 years of age, and up to three-times higher in PD patients compared to healthy individuals [35].
3. Eradication of H Pylori infection using combined antibiotic therapies can improve bioavailability and pharmacokinetics of levodopa and drug bioavailability by increasing its absorption by 21 to 54%, despite one single-centre negative study. The latter study, however, did not address blood levels of levodopa and instead focused on quality of life and motor scores [36]. Any patient with delayed time to ‘ON’ after oral levodopa absorption, as well as upper gastrointestinal symptoms of heartburn, bloating, and reflux, must have H Pylori infection tested and, if positive, be treated [37].
4. Severe constipation may arise from chronic dehydration and impacted faeces. This also interferes with oral drug absorption and a simple abdominal X-ray may show dilated bowel loops and impacted faeces [38,39]. Treatment with regular laxatives and even an enema may then be warranted, as part of the vitals, in relevant cases.

Finally, there is the issue of comorbidity- and medication-related enablers of health, such as impulse control disorders (ICD) as well as medication management. Diabetes mellitus has been proposed to be a risk factor of PD and comorbid diabetes can affect PD [40,41,42]. Consequently, blood glucose is often listed, along with urate, as associates in the revised MDS criteria for PD, while antidiabetic drugs are being examined for possible neuroprotection in PD [43]. Diabetes is a risk factor for worsening neurodegeneration, delayed gastric emptying as well as cognitive dysfunction and, hence, should be actively listed in the dashboard [44]. Other important co-morbidities, which have been proposed as risk factors for PD, also include REM Sleep behaviour disorder (RBD), with 80% of RBD patients developing neurodegenerative diseases, such as PD [45,46]. Development of PD Dementia (PDD) has been proposed to be greater in those with higher UPDRS scores, male gender, have hypertension, and, most commonly, have a history of neuropsychiatric disorders [47]. As such, greater emphasis should be on managing cognitive and psychological disorders in PwP given the risk of significant progression in PD that can occur in these cohorts; as such, the dashboard includes MoCa and MDS NMS, both of which aid in the surveillance of the emergence and presence of psychiatric and other neurological comorbidities.

**Figure 2:**
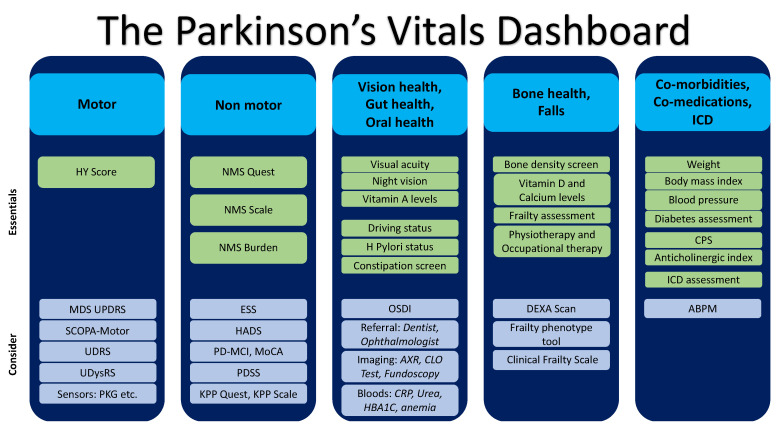
A proposed “Parkinson’s vitals dashboard” comprising the vitals and some specific measures that should be undertaken. Divided into the *essentials* which should be performed annually at review and *consider* some optional investigations and assessments if time permits. ABPM ambulatory blood pressure monitoring; AXR abdominal X-ray; CLO campylobacter-like organism test; CPS co-morbidities polypharmacy score; CRP C-reactive protein; DEXA dual energy X-ray absorptiometry scan; ESS Epworth Sleepiness Scale; HADS Hospital anxiety and depression scale; HY Hoehn and Yahr, H Pylori helicobacter pylori; ICD impulse control disorder; KPP King’s Parkinson’s Pain; MCI mild cognitive impairment; MDS-UPDRS Movement disorder society unified Parkinson’s disease rating scale; MoCA Montreal Cognitive Assessment; NMS nonmotor symptoms; NMS Quest NMS questionnaire; OSDI Ocular Surface Disease Index; PDSS PD Sleep Scale; PKG Parkinson’s kinetograph; SCOPA-motor Scales for outcomes in Parkinson’s disease motor function; UDRS unified dystonia rating scale; UDysRS unified dyskinesia rating scale.

Polypharmacy is common in PD related to comorbidities and risks side effects, which includes ICD with dopaminergic drugs, specifically dopamine agonists. Withdrawal of dopaminergic drugs, specifically dopamine agonists, also needs to follow a graded pattern to avoid dopamine agonist withdrawal syndrome [48,49]. The use of dopaminergic drugs carries with it side effects, which must be reviewed in each consultation with PwP, to ensure adequate support and holistic care are provided. Side effects include ICD, which can range from hypersexuality, gambling, binge eating, or impulsively, and other side effects, including neuropsychiatric (hallucinations, delusions) and dyskinesias [50,51]. The dashboard includes assessment of these concurrently during consultation (see Figure 2). Furthermore, specific attention needs to be given to anticholinergic drugs and a reference to the anticholinergic index of all drugs being given to PwP, as these drugs should not be used in the cholinergic subtype of PD and generally can worsen cognition and gait in PD. In this respect, a comorbidity polypharmacy score (CPS), which is defined as the sum of baseline medication and all known comorbidities, may be useful, and the severity of CPS has been traditionally stratified as mild (CPS 0–7), moderate (8–14), severe (15–21), and morbid (≥22 points). Pill burden, comorbidity, and swallowing all come into play in this respect [52,53].

## 2. Conclusions

A dashboard of the vital symptoms, which are enablers of wellness in PD, needs to be considered in every patient with PD, regardless of stage and setting, see Figure 2. The process is simple and needs to be preferably recorded on an annual basis, as part of their regular review. Attention to these vitals would ensure continuing good care for PwP and function as the cornerstone of a holistic personalised modern symptom-driven management strategy.
